# Affinity Chromatography of Native and Recombinant Proteins from Receptors for Insulin and IGF-I to Recombinant Single Chain Antibodies

**DOI:** 10.3389/fendo.2015.00166

**Published:** 2015-10-27

**Authors:** Yoko Fujita-Yamaguchi

**Affiliations:** ^1^Department of Diabetes Complications and Metabolism, Beckman Research Institute of City of Hope, Duarte, CA, USA

**Keywords:** affinity chromatography, insulin, IGF-I, receptors, single-chain antibodies

## Abstract

Affinity chromatography is an efficient method to isolate proteins by taking advantage of their affinities for specific molecules such as substrates, inhibitors, antigens, ligands, antibodies, and other interacting molecules, including subunits. Nowadays, we take the effectiveness and excellence of this technology for granted. This essay will mainly cover the use of affinity chromatography based on my experience.

## Introduction

Early in my career, I prepared and used five different affinity adsorbents by utilizing different types of interactions (Table 1) (1–5). Later in my career, I used commercially available affinity columns and beads, which specifically bind to tags or fusion proteins designed for purification of antibodies and recombinant proteins (Table 1) (6–11). Before the introduction of affinity chromatography, however, purification of a protein, which is not abundant in tissues, was not an easy task. Conventional procedures used before the affinity chromatography era included ammonium sulfate precipitation, ion-exchange chromatography, hydrophobic interaction chromatography, gel-exclusion chromatography, isoelectric focusing, etc., which gave only around five-fold purification with about a 50% recovery at each step. Depending upon how much and what kinds of contaminating proteins were in the starting materials, purification of a protein required a combination of at least four to five conventional methods, as above, that was labor intensive. Even if successful, that hard work often resulted in purification of a protein in an overall very low yield.

**Table 1:** **Examples of different affinity chromatographies**.

Reference	Affinity gels	Immobilized specific molecules	Protein purified	Fold purification
(1)	Gum arabic cross-linked gel	Branched polymers of galactose, rhamnose, arabinose, etc.	SHA (*Streptomyces* lectin)	13,300
(2)	α-Lactalbumin-Sepharose	α-LA (lactose synthetase B protein)	Galactosyltransferase (lactose synthetase A protein)	286,000
(3)	Insulin–Sepharose	Insulin	Insulin receptor	330 From WGA-Sepharose eluates
(4)	αIR3-Sepharose	Anti-IGF-I receptor mAb	IGF-I receptor	460 From WGA-Sepharose eluates
(5)	CIT-Sepharose	PDE3 inhibitor	cGMP-inhibited low Km cAMP PDE (cGI-PDE)	5,700
(6–8)	Protein A-Sepharose	Protein A	scFv–Fcs	ND
(7)	Glutathione Sepharose 4B beads	Glutathione	GST–scFvs	ND
(8–11)	Ni^2+^-Sepharose	Ni^2+^	scFvs with His-tag	ND

Affinity chromatography in general is concisely reviewed by Urh et al. (12). Drs. Cuatrecasas and Anfinsen were the first to introduce practical affinity chromatography (13, 14). Unlike conventional column chromatography, an enzyme was purified by passing it through a column containing a cross-linked polymer (or gel) to which a specific competitive inhibitor of the enzyme was covalently attached (13). All proteins without substantial affinity for the bound inhibitor passed through the column, whereas one that recognized the inhibitor bound to or was retarded in proportion to its affinity constant. Elution of the bound enzyme was readily achieved by changing conditions contributing to the binding such as a salt concentration and/or pH, or by the addition of a competitive inhibitor in a buffer solution.

In the original publication (13), the general principles and potential applications of affinity chromatography were well demonstrated by purification of *Staphylococcal* nuclease, α-chymotrypsin, and carboxypeptidase A. The solid matrix used in these studies was Sepharose (agarose, a “beaded” form of cross-linked dextran with a highly porous structure), which is still widely used for commercially available affinity columns. Activation of the Sepharose by treatment with cyanogen bromide results in a derivative that can be readily coupled to unprotonated amino groups of specific molecules having high affinities for the proteins of interest. The resultant specific molecule-coupled Sepharose is a highly stable structure which has nearly ideal properties for selective column chromatography (14). A similar approach to this was applied to purification of cGMP-inhibited low Km cAMP PDE (cGI–PDE) (Table 1) (5).

## Affinity Chromatography Experiments Prior to Purification of the Insulin Receptor

As a graduate student at the University of Tokyo in the mid 1970s, after struggling with conventional purification procedures, I was introduced to affinity chromatography. I prepared affinity adsorbents by cross-linking commercially available gum arabic, a product of acacia trees. Its structure is still unsolved, but believed to be a branched polymer of galactose, rhamnose, arabinose, and glucuronic acid with an average molecular mass of 250,000. Since my previous study revealed that the hemagglutination activity of *Streptomyces* hemagglutinin (SHA), a lectin secreted by *Streptomyces* sp. 27S5, was inhibited by l-rhamnose, d-galactose, l-arabinose, and plant polysaccharides, including gum arabic (15), gum arabic was cross-linked by epichlorohydrin according to the method for preparation of Sephadex. The solidified gum arabic was broken into small pieces, which were washed thoroughly. The gum arabic polymer gels were used to purify SHA from 15 l of culture supernatants of *Streptomyces* sp. 27S5 using a one-step batch-to-column chromatography achieving 13,300-fold purification with a yield of 60 mg and 64% recovery of the total activity (1). This was quite an accomplishment when compared to the conventional five step-purifications performed prior to the affinity purification, which resulted in only 190-fold purification with 0.006% recovery of the total activity from the starting material (15). We found in 2014 that the purified SHA kept frozen since 1974 was intact with respect to molecular mass and hemagglutination activity. New structural and functional studies are now in progress (16).

Second, quite a unique affinity chromatography was carried out to purify UDP-Gal:GlcNAc β4-galactosyltransferase from human sera (2). It had been known that lactose synthetase is composed of two protein components; UDP-Gal:GlcNAc β4-galactosyltransferase (A protein) and α-lactoalbumin (α-LA) (17). By taking advantage of subunit interactions for the affinity column design, α-LA was mixed with cyanogen bromide-activated Sepharose which yielded 6.1 mg α-LA/ml of gel (2). α-LA binding to A protein changes the acceptor specificity of A protein, which recognizes glucose as an acceptor, thus allowing the synthesis of lactose. Therefore, galactosyltransferase (A protein) was purified by applying human sera to α-LA-Sepharose in the presence of Mg^2+^ and GlcNAc, which allows A protein binding to α-LA in the column. Galactosyltransferase (A protein) was eluted from the column by the buffer lacking GlcNAc. From 155 g of total serum proteins, 220 μg of galactosyltransferase was purified by two α-LA-Sepharose chromatographies. The first column (2.5 cm × 12 cm) was used to bind the enzyme from 3.4 l of sera, whereas the reduced-size second column (0.5 cm × 10 cm) was used to concentrate and thoroughly remove non-specific proteins. The overall 286,000-fold purification was achieved with 40% recovery of the total activity (2). This work was done during my post-doctoral period as a result of a spin-off of the assignment given by my mentor.

## Purification of Biologically Active Insulin Receptor: The Same Insulin–Sepharose Column Had Been Used Over 250 Times to Purify IR with Full Binding and Tyrosine Kinase Activities

When I started to become an independent researcher at City of Hope, Dr. Keiichi Itakura and I wrote an NIH grant in which two aims were proposed. Aim 1 was to purify human insulin receptor (IR) and Aim 2 was to clone IR cDNA. By that time, I was a well-qualified protein chemist able to carry out Aim 1 whereas Dr. Itakura was very successful in manually synthesizing oligonucleotides long before automated DNA synthesizers became commercially available. Aim 2 was to use mixed oligonucleotides designed based on amino acid sequences of IR peptides as probes to clone cDNA encoding IR mRNA. To make a long story short, we were successful in purifying and obtaining amino acid sequences of IR peptides, but not in screening of cDNA encoding IR by the proposed mixed oligonucleotide probes. As it turned out, our strategy of using mixed oligonucleotides consisting of all possible codon combinations of amino acid sequences which inevitably contained 8 or 16 mixtures resulted in high levels of non-specific hybridizations to messages much more abundant than IR. By contrast, Dr. Axel Ulrich’s team at Genentech was successful in screening IR cDNA by using a long single DNA probe, which was designed based on codon usage. They also successfully used such a long probe consisting of 78 nt for the cloning of IGF-I receptor, which we purified and provided them for determination of N-terminal and peptide sequences (18).

We purified human IR from placenta by insulin affinity chromatography with new elution conditions modified from published protocols (3). Porcine insulin was conjugated to Sepharose according to the methods previously published by Cuatrecasas (14, 19). Insulin affinity chromatography had been carried out before us to purify IR. The resulting preparations were, however, not proven to be adequately pure but rather considered as a partial or incomplete purification because they did not reach a theoretical specific activity of 20 pg of insulin bound per milligram of protein when assuming IR with Mr = 300,000 binds one molecule of insulin. For example, Jacobs et al. reported the purification of IR from rat liver by insulin affinity chromatography in 1977 (20), but specific activity was 2.4 pg of insulin bound per milligram of protein. Siegel et al. purified human placental IR with a specific activity of 5.1 pg of insulin bound per milligram of protein using DEAE-cellulose chromatography and insulin–Sepharose chromatography (21). Such low specific activities could be due to contaminants in the purified receptor preparations (not yet completely purified) or to inactivation of the receptor (pure but having lost its activity). The major drawback of the insulin–Sepharose chromatography procedures introduced by Jacobs et al. (20) was that insulin-binding activity could be inactivated during elution from the column with 50 mM acetate buffer, pH 6, containing 4.5M urea.

We used two affinity columns to purify IR from human placenta. The first step was to purify glycoproteins from Triton X-100 solubilized placenta membranes by WGA-Sepharose chromatography. WGA-Shepharose bound glycoproteins contained IR as well as IGF-I receptor. As the second step of purification, the glycoprotein fractions were applied to the insulin–Sepharose column, which had been equilibrated with 50 mM Tris–HC1 buffer, pH 7.4, containing 1M NaCl, 0.1% Triton X-100, and protease inhibitors. The column was washed extensively with this buffer. The column was eluted with 50 mM acetate buffer, pH 5, containing 1M NaCl, 0.1% Triton X-100, and protease inhibitors.

The purified receptor had a high specific activity of 28.5 pg of insulin bound per milligram of protein, confirming apparent homogeneity of the preparation (3). Since the reviewer asked us to compare the two elution conditions, a half amount of WGA-Sepharose eluates was applied to the insulin–Sepharose column from which IR was eluted by 50 mM acetate buffer, pH 6, containing 4.5M urea and the other half was applied to the same column from which IR was eluted by our high salt (1M NaCl) and low pH (pH 5) elution conditions. The comparison of the elution profiles of insulin binding activity [Figure 1 and Table 2 in Ref. (3)] clearly demonstrated that IR eluted by the previously used urea-containing buffer gave a much smaller activity peak that was about one-fifth of the insulin binding activity as compared to the receptor eluted with the new conditions. This experiment demonstrated that IR was inactivated by 4.5M urea included in the elution buffer.

Our purified IR exhibited a theoretically reasonable number of binding sites and an intact β subunit which exhibited tyrosine kinase activity (3, 22). Previous studies demonstrated by cross-linking ^125^I-insulin to membrane bound IR and subsequent SDS-PAGE that IR contained α, β, and β1 subunits (23). It was later found that the β1 subunit lacks the cytoplasmic kinase domain due to proteolytic degradation during preparations of IR. Furthermore, most of the purified IR reported earlier significantly lacked an intact β subunit although it contained α and β1 subunits, whereas our purified IR contained a high level of the intact β subunit in addition to the stable α subunit. Peptide mapping of purified β and β1 subunits directly demonstrated that the β1 subunit was a part of the β subunit (24).

Around the time we purified IR, newly found IR kinase activity was being characterized using cell homogenates and partially purified IR mainly by Dr. Masato Kasuga in Dr. Ronald Kahn’s laboratory (25, 26). Our collaboration started right away to characterize IR kinase using the purified IR. I had some doubt about the presence of an intrinsic kinase in IR since it is possible that a trace amount of protein kinases could be a contaminant in the purified IR. In fact, in the case of proteases, we found that endogenous proteases were contaminated and gradually digested the IR β subunit during storage or freezing and thawing of the purified receptors (27). During such a degradation process, the β subunit lost kinase activity, revealing that the β1 subunit no longer carried the kinase domain. This observation was consistent with the fact that the β subunit could be easily degraded to the core β1 subunit during IR preparations.

The insulin–Sepharose column (2 cm × 8 cm) was reused repeatedly. The column was kept in the elution buffer and equilibrated just before using. We used the same column to purify IR 283 times. This is really remarkable since affinity columns usually lose ligands after using 10–20 times. The IR carried both insulin binding and kinase activities even until the last preparation in 1991. Why we purified IR that many times is a very good question. The preparations were used not only for amino acid sequence determination for the original cloning project but also for characterizing specific binding and kinase activity as well as interaction with other signaling molecules. Together with the purified IGF-I receptor, the purified IR had provided excellent material to definitively determine intrinsic natures of these receptors, which could not have been achieved by using impure materials containing both receptors.

## IGF-I Receptor was Purified from Passed-Through Fractions of the Insulin–Sepharose Column by an Anti-IGF-I Receptor mAb-Sepharose Column 80 Times

Nowadays, we take for granted that recombinant technology provides us with most proteins of our interests. During 1983–1985, however, IGF-I was not commercially available unlike porcine insulin. Porcine insulin has the same affinity for human IR as human insulin so that there was no problem with its supply for preparing the insulin–Sepharose column and assaying insulin binding activity. In the case of IGFs, the situation was quite different from insulin. Only three laboratories in the world were purifying IGFs from human sera. Thus, purification of the human IGF-I receptor was carried out in collaboration with Drs. Jacobs and Cuatrecusas who provided us with not only IGF-I but also anti-IGF-I receptor mAb called αIR-3 (28).

After the first step of purification by WGA-Sepharose, glycoprotein fractions were applied to the insulin–Sepharose column. Unbound passed-through fractions from the insulin–Sepharose column, which should have contained IGF-I receptor were kept frozen. The frozen WGA-Sepharose eluate prepared from a total of 21 placentas was applied to a 2-cm × 6.4-cm αIR-3 Sepharose column (0.5 mg of antibody/ml of gel) equilibrated with 50 mM Tris–HC1 buffer, pH 7.4, containing 1M NaCl, 0.1% Triton X-100, and protease inhibitors. The column was washed extensively with this buffer. The receptor was eluted with 50 mM acetate buffer, pH 5, containing 1M NaCl, 0.1% Triton X-100, and protease inhibitors, and then with 1M glycine buffer, pH 2.2, containing 1M NaCl, 0.1% Triton X-100, and protease inhibitors. Fractions (2 ml) were collected in tubes containing 3 ml of 0.5M Tris to neutralize the eluates. Fractions containing IGF-I binding activity appeared in the pH 5 elution (Fraction I) and the pH 2.2 elution (Fraction II). Fraction I contained an active IGF-I receptor but a low level of the receptor, whereas Fraction II contained substantial amounts of the receptor with reduced IGF-I binding activity (4). Fraction I was used for binding and kinase assays while Fraction II was used for amino acid sequence determination which led to cloning of the cDNA encoding human IGF-I receptor in collaboration with Drs. Ullrich and Ramachandran at Genentech as mentioned above (18). We used the same αIR-3 Sepharose column to purify IGF-I receptor 80 times. This is also a significant achievement.

## Affinity Chromatography for Recombinant Proteins

Addition of an affinity tag is an effective method for differentiating recombinant proteins expressed in bacterial and eukaryotic expression systems from the endogenous cellular proteins. The tags can be utilized for purification as well as for detecting specifically tagged recombinant proteins. The historical basis for the development of affinity tags and how to choose an appropriate affinity tag for a particular purpose are summarized in reviews (29, 30).

A variety of affinity columns combined with expression vectors designed to express proteins of interest with affinity tags are now commercially available. Protocols are provided by manufacturers, and thus it looks easy to express and purify recombinant proteins from cDNAs encoding proteins of interest. It requires, however, experience in purification and modification of the protocols to achieve the best outcome. Here are a few examples of those which we have worked out.

## Protein A-Sepharose Chromatography

To purify recombinant single chain antibodies (scFvs), we used expression vectors (not commercially available) to produce human IgG1Fc-conjugated scFv in mammalian cells (6–8). Culture supernatants collected from αIGF-IR–scFv-Fc-expressing NS0 cells were adjusted to pH 8.0 by adding 1/20 volume 1.0M Tris (pH 8.0), and passed through a protein-A Sepharose column (1 ml). The column was washed with 10 column volumes of 100 mM Tris–HCl buffer, pH 8.0. αIGF-IR scFv-Fc was eluted from the column with 100 mM glycine buffer, pH 3.0, and collected in 1.5-ml conical tubes containing 1/10 volume of 1M Tris, pH 8.0 (6).

Anti-carbohydrate (Man3) 5A3 and (Le^X^) 1F12 scFv-Fcs were similarly purified with slight modifications to accommodate the need for each preparation (7, 8). If cells can be adapted to growth in SFM, then contamination of bovine IgGs from FCS can be avoided (7). If cells were grown in media containing FCS, scFc-Fcs and bovine IgGs would be eluted together using pH 3.0 buffer. Thus, contaminating bovine IgG had to be eluted from the column with pH 5.0-buffer before elution of scFv-Fc proteins by pH 3.0 buffer (8).

## Expression and Purification of scFv Proteins in *E. coli*

Production of soluble scFvs in *Escherichia coli* requires proper disulfide bond formation, which is not easy to achieve. To circumvent this problem, fusion proteins, which can be expressed as soluble forms in *E. coli* are often used. The glutathione *S*-transferase-tag is one good example. It was used to purify anti-carbohydrate scFvs from *E. coli* crude homogenates by glutathione-affinity column chromatography (7).

The most useful tag for purification of recombinant proteins was and is the poly-histidine tag (His-tag) (29, 31). His-tag consisting of six His interacts with a transition metal ion, such as Ni^2+^ and Co^2+^, which is the basis for immobilized metal-affinity chromatography (IMAC). As described above, in bacterial cells, recombinant proteins usually fail to fold properly and accumulate as insoluble particles called inclusion bodies. Inclusion bodies are considered to be formed by non-specific hydrophobic interactions between irregularly deposited polypeptides. Those protein aggregates could provide a good reservoir of sufficient amounts of functional scFv proteins if highly efficient refolding of denatured scFv proteins can be achieved. In our laboratory, scFv proteins were purified as denatured proteins by IMAC from inclusion bodies in the presence of 3.5M Gdn-HCl, and the scFv proteins were successfully folded using the stepwise dialysis method (8–11).

## Conclusion

Affinity chromatography remains a crucial core technology for the purification of native and recombinant proteins, and affords consistently a high level of resolution and recovery.

## Conflict of Interest Statement

The author declares that the research was conducted in the absence of any commercial or financial relationships that could be construed as a potential conflict of interest.
